# Breakthrough tuberculosis disease among people with HIV – Should we be worried? A retrospective longitudinal study

**DOI:** 10.1371/journal.pone.0211688

**Published:** 2019-02-04

**Authors:** Kesetebirhan Delele Yirdaw, Alula M. Teklu, Admasu T. Mamuye, Solomon Zewdu

**Affiliations:** 1 FHI360, Addis Ababa, Ethiopia; 2 Monitoring Evaluation Research Quality Consultancy, Addis Ababa, Ethiopia; 3 Addis Ababa University College of Health Sciences, Addis Ababa, Ethiopia; 4 Bill and Melinda Gates Foundation, Addis Ababa, Ethiopia; University of KwaZulu-Natal, SOUTH AFRICA

## Abstract

**Introduction:**

Isoniazid preventive therapy (IPT) is a proven means to prevent tuberculosis (TB) disease among people living with HIV (PLHIV). However, there is a concern that patients often develop tuberculosis disease while receiving IPT, defined here as breakthrough tuberculosis, which may affect treatment outcome. In this study, we assessed the magnitude and determinants of breakthrough tuberculosis.

**Methods:**

A multisite retrospective longitudinal study from the year 2005 to 2014 involving 11 randomly selected hospitals from the Addis Ababa, SNNPR (Southern Nations Nationalities and Peoples Region), and Gambela regions of Ethiopia was carried out to assess the occurrence of breakthrough tuberculosis. Cox regression analysis was used to study factors associated with breakthrough TB.

**Results:**

4,484 patients in chronic HIV care received IPT. Eighty percent of the same number received antiretroviral therapy (ART). Tuberculosis developed in 88 of 4,484 (2%) patients of which 24 (0.5%) were diagnosed while receiving IPT. Breakthrough TB incidence was 1106 per 100,000 person-years (PY) (95% CI: 742–1651) while TB incidence after completing IPT was 624 per 100,000 PY (95% CI: 488–797). Seven of the 24 (29%) breakthrough TB cases were diagnosed within the first month of IPT initiation. Of 15 patients who developed breakthrough TB while on ART, nine (60%) were diagnosed within the first six months of ART initiation. Having high CD4 cell count and being on ART were associated with having lower risk of developing TB and breakthrough TB.

**Conclusion:**

Breakthrough TB was uncommon in the study setting. Even then, taking ART reduced the risk of its occurrence. Slightly more than a quarter of the cases of breakthrough TB occurred in the first month of treatment and may be existing undiagnosed TB cases which were missed during diagnostic work-up.

## Introduction

The use of Isoniazid preventive therapy (IPT) which results into lower risk of progression to TB disease and TB related mortality for people living with Human Immunodeficiency Virus (HIV) has been found to be effective in treating latent tuberculosis (TB) infection (LTBI). [1–6] Effectiveness of IPT with or without ART has been confirmed by many observational studies in low and middle-income settings. [5–8] In fact, concomitant use helps to reduce the occurrence of TB disease soon after ART initiation which is when the risk is higher. [2,9]

IPT initiation in Ethiopia for people living with HIV during 2013 was at 4.8% which is low. [10] This indicates the inadequacy of implementation of this life saving intervention despite the proven effectiveness. There are several barriers that impede IPT implementation. Medication related barriers which are likely to account for a small fraction of reasons why medication is discontinued or deferred include potential pill burden, side effects, and overlapping side effects with ART medications. [11] The most important factor, however, is the attitude of health care workers towards IPT provision related to concerns with uncertainty in ruling out TB disease, and providers’ fear of patients developing TB disease while taking IPT which they believe lead to drug resistance. [12–14] The evidence, however, indicates that most of these worries are not justified. For instance, one of the feared toxicities is hepatotoxicity and the incidence of clinically significant elevation of liver enzymes is only 6 per 1000 people, which is low. [15] Assessing the risk of Isoniazid drug resistant following IPT use, a meta-analysis of 36,000 patients who took IPT indicated that the risk of emergence of Isoniazid resistance was very low and not statistically significant. [16] Furthermore, the WHO (World Health Organisation) symptom based tuberculosis screening algorithm was found to have negative predictive value of >95%, making it an effective tool to rule out TB disease in PLHIV. [11,17]

While these evidences are adequate to reassure clinicians that IPT use is safe, it is important to find out about the risk of developing TB disease during and after taking IPT to identify and address major risk factors associated with it so as to provide evidence based guidance to health care workers and policy makers. In this study, we describe the incidence of TB disease while taking IPT (defined here as breakthrough TB) and after its completion, as well as factors associated with it among PLHIV receiving chronic HIV care. As a secondary outcome we also describe effect of TB disease on retention in care.

## Methods

### Study design, population, and setting

**Fig 1:**
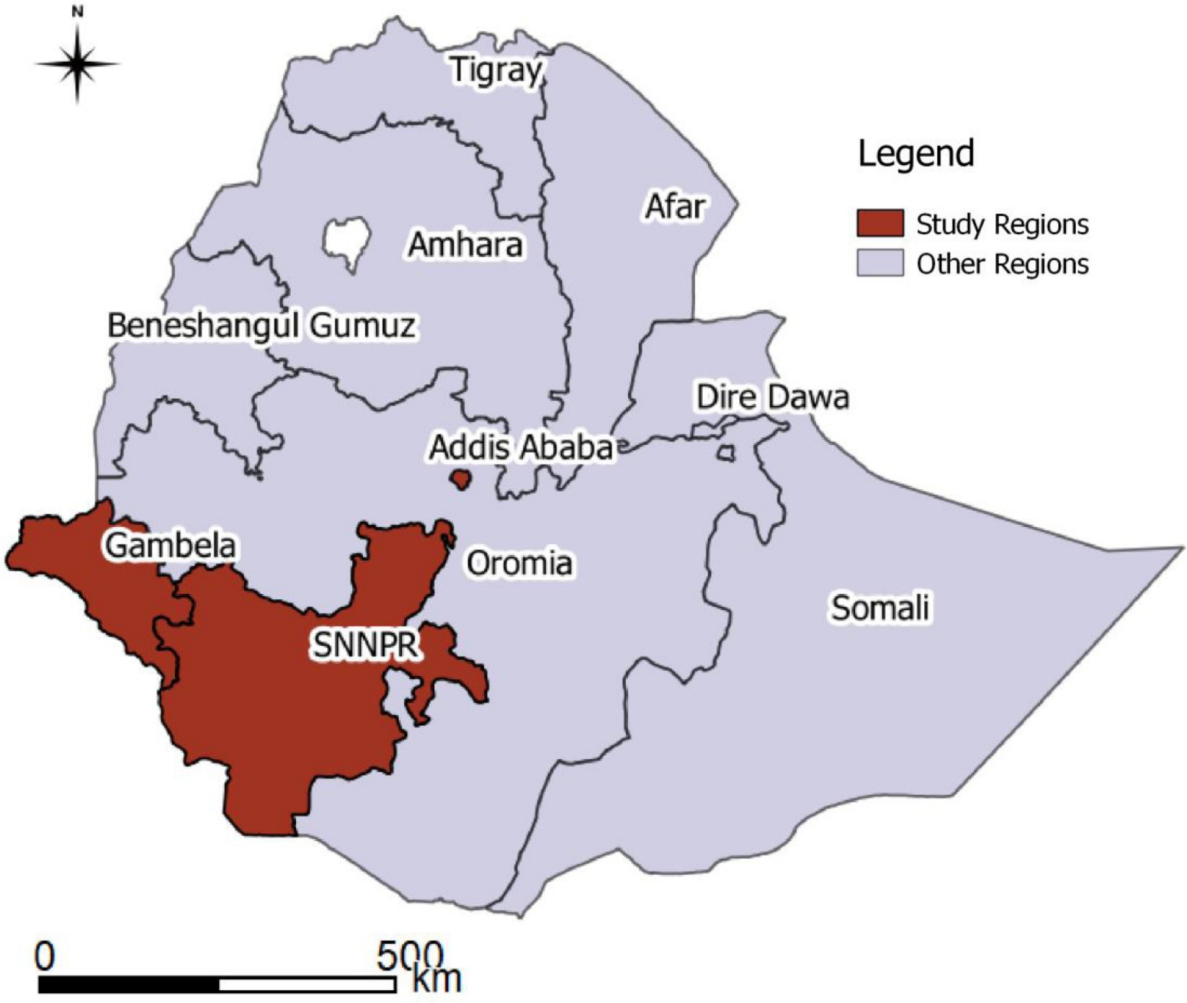
Map Indicating Regions where Selected Health Facilities are located.

This is a retrospective, observational, longitudinal study that used existing records of patients in 11 randomly selected hospitals (out of 35) located in three regions of Ethiopia; namely, Addis Ababa, Gambella, and Southern Nations Nationalities and Peoples (SNNP) Region (Fig 1). Addis Ababa, which is the capital city of Ethiopia, is totally urban while only 15% of the other regions are urban. The regions were selected because of similarity in their data management system and the authors were familiar with it. Two of the regions, Addis Ababa and Gambella, are high HIV burden/prevalence regions with adult HIV prevalence of 3.4% and 4.8% respectively according to the 2016 HIV prevalence estimates. In the SNNP region, only urban areas where all hospitals are located are with high prevalence rate. Adult prevalence in SNNP region was 0.4%. [18]

All government hospitals have been providing chronic HIV care (including ART) and IPT for eligible clients based on the national ART and TB/HIV guidelines since 2005. Eligibility for ART was based on the following criteria: all WHO stage IV clients, WHO stage III clients with CD4≤350 and WHO stage I or II with CD4≤ 200. In the absence of CD4 testing, all WHO stages III clients were eligible for ART. The first line ARV regimens used were according to national and international guidelines. [19,20]

HIV infected clients were screened for TB disease during each clinic visit using WHO recommended symptom-based screening, and symptomatic patients were evaluated by diagnostic tests that included clinical evaluation, sputum smear microscopy, as well as chest radiography. Anti-tuberculosis treatment was provided when TB was diagnosed and IPT was given to those without TB disease depending on availability of Isoniazid and absence of contraindications. Therefore, it means IPT is recommended for all PLHIV not diagnosed with TB disease. Tuberculin skin test (TST) was not available and was not required to start IPT as per treatment guidelines. IPT was provided at a dosage of Isoniazid 300 mg/day for adults and 5–10 mg/kg/day for children for a period of six months. Patients collected their drugs every month until the treatment was completed. Patients on IPT were monitored at every visit for development of active TB disease, adherence to treatment and adverse drug reactions. [21] Health care workers and data clerks for keeping records of clinical care were trained and supervised so as to maintain accurate records. [22]

### Study period, population and sampling

The sampling frame for selecting the hospitals constituted 35 hospitals from the three regions. STATA statistical software Version 12 was used to randomly select 11 hospitals. Sample size was estimated based on simple proportion formula taking incident TB disease after starting IPT 1.5% based on study in a similar setting. [9] Taking the level of precision to be 0.5%, confidence interval 95%, and estimated loss of 20%, the sample size was 4,099. Therefore, all newly enrolled PLHIV in chronic HIV care who started IPT in these randomly selected hospitals between September 2005 and October 2013 were studied.

### Exposure and outcome variables

The exposure variables of interest are: age, gender, baseline WHO stage, baseline CD4 count, ART treatment status at last observation, and duration on ART.

The primary outcome variable was incident TB disease after starting IPT. The occurrence of TB disease while receiving IPT was defined as breakthrough TB. Incident TB disease that occurred after IPT completion was defined as ‘TB after IPT completion’. ‘Those who took IPT but did not develop TB disease were classified as ‘no TB’.

The secondary outcome variable was retention in care, described as final status at the end of the study. Those retained in care (being active on ART treatment at the end of last observation or in April 2014 were considered as having a favourable outcome. Those who died, stopped treatment or were lost to follow-up despite adequate tracing to locate them, were categorized as having an unfavourable treatment outcome.

### Data analysis

MS Access 2007 was used to store and clean the data. STATA statistical software Version 12 was used to do descriptive and analytic analysis. (Look at supporting information S1 Table for the final dataset) Proportions were used to describe exposure and outcome variables. To calculate TB disease incidence, person-time at risk of TB disease was accrued from the date of IPT initiation until appearance of incident TB disease, death, transfer to other facility, loss to follow-up (LTFU), or last visit. Any patient who failed to come for care for over a month and could not be located by treatment supporters was classified as LTFU. Since there was no data on IPT completion, intention-to-treat analysis was used assuming IPT treatment was completed. TB incidence was measured by splitting time every six months. Determinants of incident TB disease were analyzed using Cox regression analysis. Interaction terms were used to determine if risk factors of developing TB while taking IPT was different from that of developing TB after completing IPT. Those variables with p value <0.25 were included in the final model. P value <0.05 was used to determine statistically significant associations in the final model. Finally, determinants of retention in care were identified by studying time to unfavourable final status using Cox regression following the same step as described earlier. Person-time was accrued from time of IPT initiation to occurrence of final status. Health facilities’ ID was used to control for clustering by including it as stratifying factor in the regression models.

### Ethics

Ethics approval for the study was obtained from the National Research Ethics Review Committee (NRERC) of Ethiopia (reference number 3.10/712/04). Consent forms for individual clients were not required since an existing de-identified and de-linked database was used.

## Results

Of 35,789 patients enrolled for treatment in the study sites, 4,484 (12.5%) received IPT. Of those that received IPT, the majority, 79.8% (n = 3,578), also received antiretroviral therapy. The median age of the study sample was 30 years (interquartile range 25–37). Children below 15 years of age constituted 10.8% (n = 483). Female gender accounted for 63.6% (n = 2,854). Most patients (60.7%, n = 2,724) were in WHO stage III or IV at enrolment. The median baseline CD4 cell count was 199/mm^3^ (interquartile range 124–316). (Table 1) Patients were followed for a median of 2.9 years (inter-quartile range: 1.6–3.9 years).

**Table 1:** Baseline Demographic and clinical characteristics of study patients on isoniazid preventive therapy in Ethiopia, September 2005 to October 2013 (n = 4,484).

Variable	Sub-category	Breakthrough TB (Row %)	TB after IPT completion, (Row %)	Total
**Age**	<15	2 (0.4%)	4 (0.8%)	483
** **	≥15	22 (0.5%)	60 (1.5%)	4001
**Gender**	Female	13 (0.5%)	37 (1.3%)	2854
** **	Male	11 (0.7%)	27 (1.7%)	1630
**Baseline WHO Stage**	I or II	8 (0.5%)	24 (1.4%)	1760
** **	III or IV	16 (0.6%)	40 (1.5%)	2724
**Baseline CD4 Count**	<100	5 (0.6%)	13 (1.6%)	801
** **	100–349	13 (0.5%)	43 (1.6%)	2670
** **	≥350	6 (0.6%)	8 (0.8%)	1013
Treatment Status*	IPT only	9 (1.0%)	20 (2.2%)	906
** **	ART and IPT	15 (0.4%)	44 (1.2%)	3578
**Grand total**		24 (0.5%)	64 (1.4%)	4484

*Chi^**2**^***P* value = 0.009;** *P* value for other variables is >0.05.

### Incident TB disease

**Fig 2:**
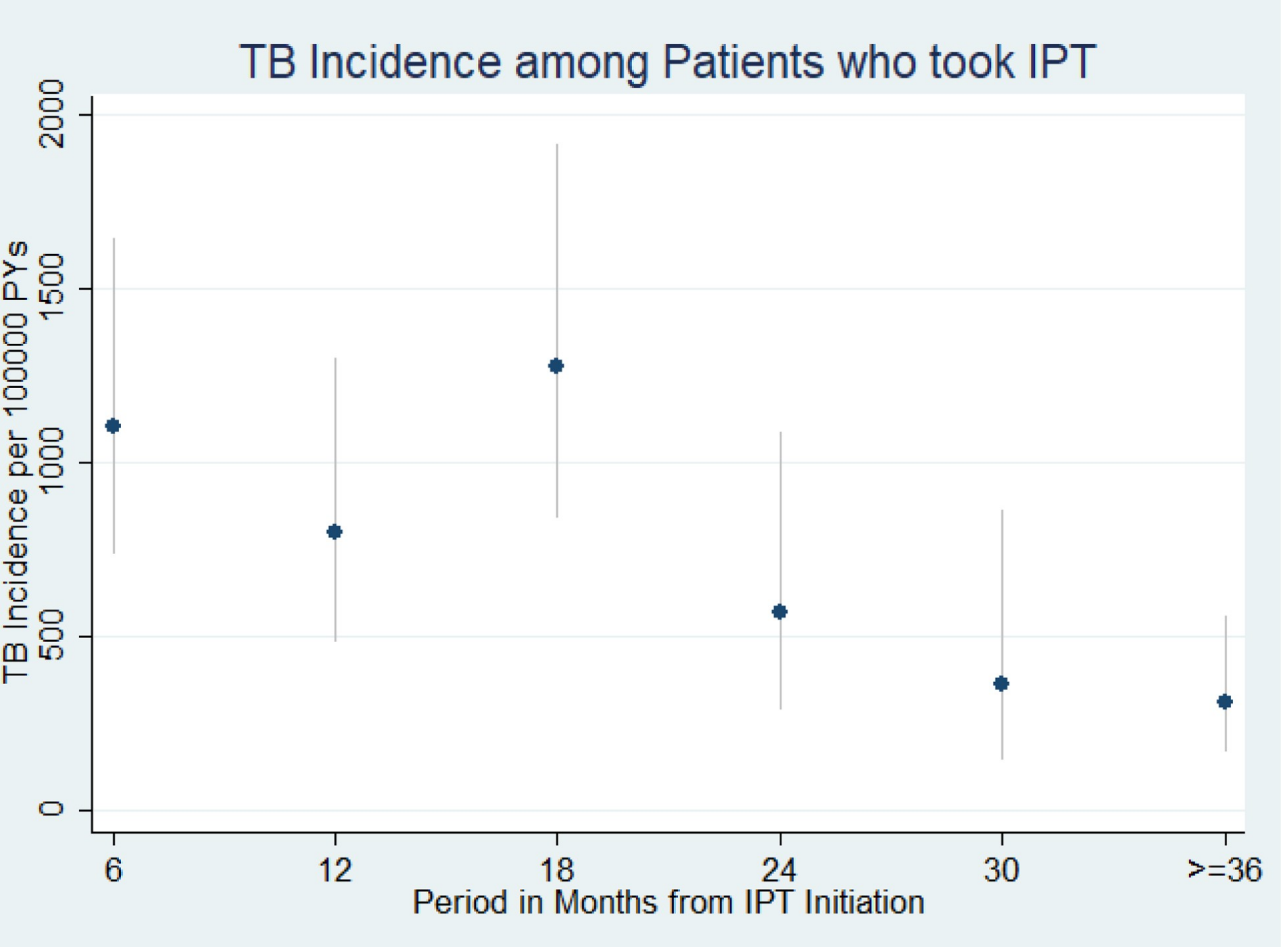
TB Incidence among patients who took isoniazid preventive therapy in Ethiopia, September 2005 to October 2013 (n = 4,484). TB incidence is indicated by dots while the spike indicates confidence interval. TB incidence was high in the first one year. It declined sharply then after.

Of 4,484 patients, 88 developed TB (2%). Of these, 24/88 (27.3%) patients were diagnosed with TB while receiving IPT (breakthrough TB). TB incidence was 708 per 100,000 person-years (PY) (95% CI: 575–873). As shown in Fig 2, TB incidence was high in the first 18 months. The incidence of breakthrough TB was 1106 per 100,000 PY (95% CI: 742–1651) while TB incidence after completing IPT was 624 per 100,000 PY (95% CI: 488–797). Seven of 24 (29%) breakthrough TB cases were diagnosed within the first month of IPT initiation.

### Determinants of incident TB disease among patients that took IPT

Table 2 below shows that those with higher baseline CD4 cell count (≥350 cells/mm^3^) (adjusted Hazard ratio (95% confidence interval): 0.12 (0.05–0.28)) and those receiving ART (adjusted Hazard ratio (95% confidence interval): 0.08 (0.04–0.14)) were less likely to develop tuberculosis disease. Other variables did not have an effect on the occurrence of incident TB disease among PLHIV who took IPT. Looking at effect modification by follow-up period, no evidence was identified for difference in risk factors for development of TB disease during or after IPT completion. (Table 3)

**Table 2:** Determinants of occurrence of TB disease in study patients in Ethiopia that used isonizid preventive therapy, September 2005 to October 2013 (n = 4,484).

Variable	Sub-category	Person Years	TB	Unadjusted Hazard Ratio (95% CI), P value	Adjusted Hazard Ratio (95% CI), P value
**Age**	<15	860	6		
** **	≥15	11570	82	0.88 (0.36–2.17), 0.789	
**Gender**	Female	8010	50		1
** **	Male	4420	38	1.32 (0.86–2.01), 0.199	1.37 (0.90–2.10), 0.143
**Baseline WHO Stage**	I or II	5200	32		
** **	III or IV	7220	56	1.23 (0.79–1.92), 0.354	
**Baseline CD4 Count**	<100	2490	18		1
** **	100–349	8220	56	0.73 (0.42–1.24), 0.245	0.65 (0.37–1.12), 0.120
** **	≥350	1720	14	0.83 (0.40–1.72), 0.621	**0.12 (0.05–0.28),<0.001**
**Treatment Status**	IPT only	1240	29		1
** **	ART and IPT	11190	59	**0.25 (0.16–0.41),<0.001**	**0.08 (0.04–0.14),<0.001**

**Table 3:** Determinants of occurrence of TB disease in study patients in Ethiopia during and after isoniazid preventive therapy use, September 2005 to October 2013 (n = 4,484).

		While taking IPT			After Completing IPT			
Variable	Sub-category	Person Years	TB	Hazard Ratio (95% CI)	Person Years	TB	Hazard Ratio (95% CI)	P value*
**Age**	<15	240	2		620	4		
** **	≥15	1930	22	0.91 (0.17–4.81)	9640	60	0.9 (0.3–2.66)	0.799
**Gender**	Female	1380	13		6630	37		
** **	Male	790	11	1.45 (0.65–3.25)	3630	27	1.27 (0.77–2.09)	0.794
**Baseline WHO Stage**	I or II	870	8		4340	24		
** **	III or IV	1300	16	1.2 (0.51–2.84)	5920	40	1.24 (0.74–2.07)	0.909
**Baseline CD4 Count**	<100	390	5		2090	13		
** **	100–349	1310	13	0.58 (0.2–1.65)	6910	43	0.79 (0.42–1.48)	0.720
** **	≥350	460	6	0.71 (0.21–2.43)	1250	8	0.77 (0.31–1.92)	0.975
**Treatment Status**	IPT only	400	9		840	20		
** **	ART and IPT	1770	15	0.51 (0.21–1.21)	9420	44	0.2 (0.11–0.36)	0.156

*Test for interaction for factor with time

Evaluating the timing of TB cases in relation to ART initiation, 60% (9/15) of breakthrough TB cases occurred in the first six months of ART initiation as compared to 23.7% (14/59) of all TB cases that occurred in the same time period.

### Retention in care

Overall 82% of patients were on ART treatment at last observation. (Table 4) Most patients with unfavourable follow-up outcome were lost to follow-up. Those who didn’t develop TB were less likely to have unfavorable final status (adjusted Hazard ratio (95% confidence interval) 0.52 (0.27–1.00)) but it was not statistically significant at the 5% level. Only being male was associated with increased hazard of having unfavourable final status (adjusted Hazard ratio (95% confidence interval) 1.47 (1.19–1.82)). (Table 5)

**Table 4:** Final status of study patients on antiretroviral therapy and isoniazid preventive therapy in Ethiopia, September 2005 to October 2013 (n = 3,578). Final status at last visit or observation in April 2014 indicated here.

Last Status	Frequency	Percentage	Cumulative Percentage
Died*	66	2	2
Lost to follow-up*	298	8	10
Stopped treatment*	2	0.06	10
**Transferred out**	293	8	18
**Active**	2919	82	100

* Died, lost to follow-up, and stopped treatment contributed to unfavourable outcome which accounted for 366 (10%) of final status.

**Table 5:** Determinants of unfavourable* final status among patients on antiretroviral therapy and isoniazid preventive therapy (n = 3,578). Those who died, were lost to follow-up, or stopped antiretroviral treatment at last observation were considered as having unfavourable outcome.

Variable	Sub-category	Person Years	Unfavorable outcome*	Unadjusted Hazard Ratio (95% CI), P value	Adjusted Hazard Ratio (95% CI), P value
**Age**	<15	640	16	1	
** **	≥15	10300	334	1.01 (0.58–1.77), 0.976	
**Gender**	Female	6960	185	1	1
** **	Male	3970	165	**1.51 (1.22–1.86), <0.001**	**1.47 (1.19–1.82), <0.001**
**Baseline WHO Stage**	I or II	4910	136	1	1
	III or IV	6030	214	1.21 (0.97–1.51), 0.090	1.16 (0.92–1.45), 0.204
**Baseline CD4 Count**	<100	2450	88	1	1
	100–349	7830	247	0.79 (0.61–1.01), 0.065	0.79 (0.61–1.01), 0.068
	≥350	660	15	0.60 (0.34–1.06), 0.079	0.60 (0.34–1.06), 0.076
**TB disease status**	TB after IPT completion	140	9	1	1
	No TB	10760	335	0.53 (0.27–1.02), 0.058	0.52 (0.27–1.00), 0.052
	Breakthrough TB	30	6	2.40 (0.85–6.77), 0.098	2.36 (0.83–6.68), 0.107

* Died, lost to follow-up, and stopped treatment contributed to unfavourable outcome which accounted for 366 (10%) of final status.

## Discussion

Breakthrough TB was uncommon among those receiving IPT in the study setting. Its incidence was 1106 per 100,000 person-years on IPT. Slightly more than a quarter of these cases were diagnosed in the first month of starting IPT. This may represent undiagnosed cases that were missed due to failure to properly adhere to WHO’s TB screening algorithm or difficulty ruling out TB using AFB and/or Chest X-ray only. The diagnostic challenge may be partially alleviated as there is an increased access to the GeneXpert test across the country, but especially in hospitals where GeneXpert tests are commonly located. [23]

Even though breakthrough TB was not specifically described in previous studies, many studies that determined effectiveness of IPT in preventing TB disease among PLHIV invariably illustrated the occurrence of TB after using IPT. Finding from a study in Ethiopia reported a lower incidence of 697 per 100,000 PY. [9] Another study came up with results much lower than the current study with an incidence of 265 per 100,000 PY. [24] A higher incidence (2,100 per 100,000 PY) of TB after IPT use was reported in studies done in three developed nations (USA, Canada, and Spain) and Brazil. In these studies TB burden was higher because most patients did not receive ART [2,25] while in the current study the reverse was the case.

TB incidence in patients that took IPT is higher (three fold higher) than that of the general population in Ethiopia which approximated at 224 per 100,000 people during 2013. [26,27] This indicates that even among PLHIV that received IPT and ART, active TB case finding by screening these at risk clients during every clinic visit is critical to detect and treat TB early. In this study those with higher baseline CD4 or on ART were less likely to develop breakthrough TB and TB after IPT completion. This is good news in a time when test-and-treat is being implemented and clients are started on ART at higher CD4 cell counts. [28,29] This study, however, also supports that the first few months after ART initiation are critical and every patient should be monitored strictly so that immune-reconstitution inflammatory syndrome (IRIS), which is another likely reason behind high occurrence of TB disease soon after ART initiation, can be detected and managed. [28] In this study, the first year was when the highest fraction of cases were detected which coincided with the period that TB IRIS was the highest. This was true for both breakthrough TB and TB diagnosed after IPT completion.

One study [30] described how WHO’s TB diagnostic algorithm is not routinely followed in very large numbers of PLHIV screened for TB in South Africa. This happened especially in TB suspects following a negative first test by AFB or GeneXpert. Further test or follow-up was performed in only 24% of individuals. This may be one reason why there were many cases of breakthrough TB cases diagnosed in the first month after IPT initiation. Further assessment is needed to understand what happened in these cases or how adherence to diagnostic algorithm in ART clinics can be improved in the future.

IPT uptake in our study setting was low at 12.5% at the end of follow-up. Of course, this includes all cases who received IPT before or after receiving ART and thus does not say anything about the timeliness of IPT provided. For PLHIV, the most important time to receive IPT is before, during, or soon after starting ART where the risk of developing TB is higher. [9] IPT uptake in the study setting is low considering the facilities are hospitals which have better access to IPT. The WHO global TB report during 2013 indicated IPT coverage of 4.8% [10] among eligible PLHIV newly enrolled in HIV care as compared to 52% in 2016 which shows significant improvement. [31]

As for the impact on retention in care, after controlling for age, gender, baseline WHO stage and CD4 count, there was no significant difference on the effect of breakthrough TB on remaining in care as compared to those having TB after IPT completion. The negative effect of TB on adherence and even mortality among PLHIV is a well-established fact and was identified in this study as well. [32]

This is a multisite study which was adequately powered and one of a few studies to describe breakthrough TB disease among PLHIV receiving IPT. There are some important limitations of this study. First, analysis was on an intention-to-treat basis since data on IPT adherence was not captured at all and IPT completion was not documented. Adherence for IPT in Ethiopia was reported to be 64% and 86% by two studies. Overall, those on ART were found to have better adherence. [33,34] In this study, most patients also received ART and thus IPT adherence may have been better. The other limitation is that this study utilized secondary data and thus limitations that arise from use of existing records apply here. Also, the findings of the study cannot be generalized to other regions in the country and hence further study with better representation of treatment sites can help to consolidate these findings.

## Conclusion

Breakthrough TB was uncommon in the study setting. A significant proportion of it occurred in the first month of treatment and could be due to difficulty in diagnosing TB disease with AFB+/-Chest X-ray or failure to strictly follow TB screening and diagnostic algorithm to rule out TB after adequate follow-up.

## Supporting information

S1 Table Study dataset. (XLS)
